# Raising Awareness of Mistreatment Policies and Reporting Procedures Using a Novel Medical Student Mobile Application

**DOI:** 10.1007/s11606-024-08948-8

**Published:** 2024-07-22

**Authors:** Seema Baranwal, Stacy B. Ellen, Carolyn Giordano, Igor Los, Arnold Smolen, Bisan A. Salhi

**Affiliations:** Drexel University College of Medicine, Philadelphia, PA USA

**Keywords:** medical student mistreatment, technology, mobile app

## Abstract

**Background:**

Reports of mistreatment are an important first step to improving medical students’ learning environment. Students may not report mistreatment due to a lack of awareness of institutional policies, reporting procedures, or for fear of reprisal.

**Aim:**

We sought to determine if a medical school cross-platform mobile application (app) could be used to improve students’ awareness of mistreatment policies and procedures.

**Setting and Participants:**

Participants in this intervention included Drexel University College of Medicine (DUCOM) medical students, faculty, and Student Affairs Deans.

**Program Description:**

We created the DUCOMpass© app to make mistreatment policies and procedures more readily available and to ease mistreatment reporting for medical students.

**Program Evaluation:**

To determine the efficacy of the app at raising mistreatment awareness, we analyzed our institutional Graduation Questionnaire data before and after the introduction of the app (from 2016 to 2023) as compared with the national average. We verified our students’ self-reported data with app usage data.

**Discussion:**

To our knowledge, this is the first instance of a medical school mobile app being implemented to successfully address medical student mistreatment awareness and reporting. We found that reaching students in a familiar and easily accessible mode(s) of communication is a catalyst for lasting change.

**NIH Trial Registry:**

Not applicable.

## INTRODUCTION

Creating a safe learning environment free of mistreatment is paramount in undergraduate medical education. Unfortunately, medical students continue to experience mistreatment and abuse during their education, especially during their clinical rotations.^1–4^ To address medical student mistreatment and its consequences (e.g., burnout, lower career satisfaction, poor mental health),^2,5–8^ the American Association of Medical Colleges (AAMC) has long recognized medical student mistreatment as a critical area for improvement in medical education.^2^ To better measure and address medical student mistreatment, the AAMC Graduation Questionnaire (GQ) has included questions about, and evidence of, mistreatment since 1991.^2^ According to the GQ, approximately 38% of medical students graduating in 2023 reported that they had experienced mistreatment at least once, a percentage largely unchanged over the past several years.^9^

An important barrier to addressing medical student mistreatment is reporting. Students may not report incidents of mistreatment due to a lack of awareness of mistreatment policies or reporting procedures, a perception that the reporting process may be too onerous, or for fear of reprisal.^10^ Raising awareness of existing mistreatment policies and creating a mistreatment reporting process that is seamless, easily accessible, and anonymous for students are critical first steps to addressing incidents of mistreatment.

Based on the 2017 GQ data, medical students at the Drexel University College of Medicine (DUCOM) had rates of awareness of the mistreatment policy and mistreatment reporting procedures lower than the national averages of all United States (US) accredited allopathic medical schools, despite being required to read and attest to understanding of our medical student handbook annually. We describe here our experiences developing a mobile application (app) to promote awareness of DUCOM’s mistreatment reporting policies and procedures.

## SETTING AND PARTICIPANTS

DUCOM is the largest private MD-granting medical school in the United States (US) with two 4-year campuses, seven regional medical campuses across the country, and nearly 1200 medical students enrolled. Our distributed training model affords students a diversity of learning environments, teaching faculty, experiences, and opportunities. Nevertheless, the variability in educational settings and institutional procedures and policies presents a challenge in making students aware of mistreatment and other policies and procedures. Participants in this intervention included DUCOM medical students, faculty, and Student Affairs Deans.

## PROGRAM DESCRIPTION

Prior to the 2017 GQ report, we relied on making our mistreatment and other policies and procedures available to students on the DUCOM website and making students aware of our policies in required class meetings. Students who wished to report mistreatment during this time were asked to reach out to their Student Affairs Deans to file a complaint. In 2017–2018, we created DUCOMpass©, a cross-platform mobile app to make mistreatment processes and procedures readily available to students. The app was developed by our Student Affairs Deans in partnership with our Technology in Medical Education Team. Initial development took approximately 8 weeks of combined effort over the course of a year, and we currently spend approximately 2 weeks per year for app maintenance. There was no additional financial support or funding involved in the development of the app.

The DUCOMpass© app was developed on the software development platform Ionic, which is built on top of an Angular technology and Apache Cordova mobile app development framework. This app is designed for iOS and Android mobile devices and for hosting on the internet as a web application. Students submit information using internally developed data entry forms and the data transits to the server via a secure web service, which resides on a web server hosted internally by Drexel University. The data submitted is securely stored in the Microsoft Structured Query Language (SQL) Server database, which can only be accessed through single sign-on by designated institutional users.

Student engagement began with a schoolwide competition to name the app. To promote awareness and use of the app, we included other important institutional policies and procedures on the app (e.g., needlestick injury reporting procedures, mental health service access instructions). Once available, students were required to download the app starting in the 2018–2019 academic year. We have reinforced this requirement during annual mandatory class meetings since the implementation of the app. With the exception of the initial “roll out period,” our engagement with students regarding mistreatment and other DUCOM policies and procedures remained unchanged.

Among other resources, the app includes an easily accessible and anonymous reporting form to promote more accurate mistreatment tracking and follow-up at our institution. Students who select the “mistreatment” field can read a statement affirming our commitment to creating a positive learning environment and providing examples of mistreatment. Students can also view the mistreatment policy and are linked to the student handbook for the next steps. They can then choose to report mistreatment through the DUCOMpass© app in one of two ways: (1) by using the electronic mistreatment reporting form (anonymously or by including their name); or (2) via direct contact with an on-call Student Affairs Dean (through a telephone hotline until winter 2021 and a text line thereafter).

Entries in the electronic mistreatment reporting form are later compiled into an institutional password-protected database accessible to select deans from the Student Affairs, Diversity, Equity, and Inclusion, and Clinical Education departments. These deans review students to verify that they constitute mistreatment and/or decide if further action is needed. In cases deemed to represent mistreatment, we proceed as follows. Students who identify themselves in the complaint are contacted by the appropriate dean to verify receipt of the complaint and to gather further information as needed. Our priority in speaking with students in the initial phase is to ensure their safety and assess their current learning environment. In the absence of acute safety or learning environment concerns, students can opt to allow further action to happen on the date of the discussion, at the end of the course and/or clinical rotation, or upon posting their grades. Next, the dean(s) responsible for addressing the report follow the protocol appropriate for the specific situation (e.g., contacting the designated institutional official of a hospital or the residency program director) for the next steps. Once the situation is investigated and appropriate action is taken (e.g., remediation of the perpetrator), the dean reports the outcome to the student.

## PROGRAM EVALUATION

**Figure 1:**
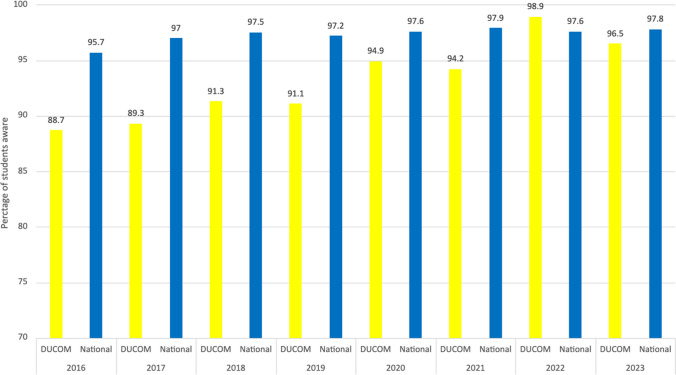
DUCOM vs. national medical students responding “yes” to the AAMC GQ question, “Are you aware that your school has policies regarding the mistreatment of medical students?” The intervention of the mobile app was introduced in the 2018–2019 academic year.

**Figure 2:**
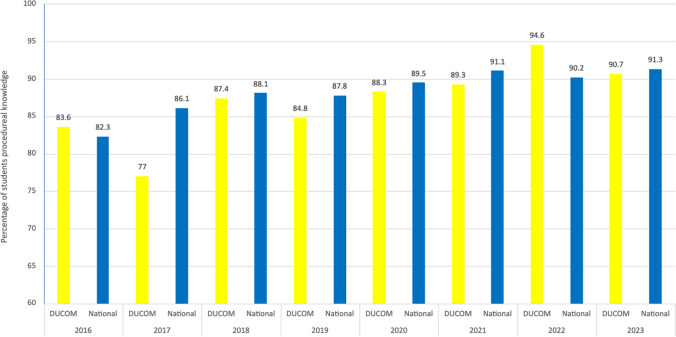
DUCOM vs. national medical students responding “yes” to the AAMC GQ question, “Do you know the procedures as your school for reporting the mistreatment of medical students?” The intervention of the mobile app was introduced in the 2018–2019 academic year.

To determine the efficacy of the app at raising mistreatment awareness, we analyzed our institutional GQ data from 2016 to 2023 as compared with the national average. After widespread use of the mobile application since the 2018–2019 academic year, the annual GQ data from 2016 until 2023 was analyzed (Figs. 1 and 2). The 2022 GQ data reflects the experience of the first class that has used the DUCOMpass© app for all 4 years of medical school. The data shows a year-over-year improvement in both the awareness of mistreatment policies and the mistreatment reporting procedures. Notably, since 2016 to 2022, there was a 10-point increase in awareness of mistreatment policies from 88.7 to 98.9% aware for Drexel University students, and an 11-point increase in knowing the procedures for reporting the mistreatment from 83.6 to 94.6%. Moreover, the 2022 DUCOM GQ shows that awareness of mistreatment policies and procedures for reporting mistreatment at our institution surpassed the national average.

To verify our students’ self-reported awareness from the GQ data, we analyzed the number of mistreatment reports submitted before and after the implementation of the app. Prior to the introduction of the app (academic years 2012–2017), students submitted an average of 15.6 reports of mistreatment per year by direct outreach to their Student Affairs Deans. Since the introduction of the app (academic years 2018–2023), students have submitted an average of 27.8 reports of mistreatment per year. Moreover, since the implementation of the app, students have rarely reported mistreatment through other avenues (e.g., a meeting with a Student Affairs Dean or the Student Affairs text line).

## DISCUSSION

To our knowledge, this is the first instance of a medical school mobile app being implemented to successfully address medical student mistreatment awareness and reporting. We found that portable accessibility of reporting was instrumental in improving the rates of awareness of mistreatment policies and reporting at our institution. Indeed, since the implementation of the app, it has become the primary mode of reporting mistreatment at DUCOM. We have also learned that the app can be used for other time-sensitive student needs. For instance, we are using the DUCOMpass© app to aid our students in accessing personal safety resources and mental health care, and to improve absence reporting and tracking. Now that the app is an established component of our medical students’ experience and the primary mode of reporting mistreatment, we are poised to expand and further support students when mistreatment occurs. Further, we plan to use the DUCOMpass© app to increase student access to mental health services, including telehealth services. While we have primarily geared the app towards students, we plan to create a faculty and staff version of the app to increase their awareness of reporting policies and procedures and to aid them in assisting students with time-sensitive matters (e.g., access to DUCOM mental health services).

One limitation of this intervention is the challenge of definitively identifying the initial causes of the low mistreatment awareness among students. It is therefore possible that increased organizational or ambient discussion of mistreatment or other confounders may have increased subjective awareness of our mistreatment policies. We are, however, unaware of any factors at DUCOM that would have contributed to students’ awareness of mistreatment policies and procedures (e.g., a sentinel event). Moreover, except for the initial introductory period, our engagement with students regarding mistreatment reporting has remained unchanged. Our students’ self-reported awareness of DUCOM mistreatment policies and procedures has increased substantially from and remained steadily above our institution’s pre-implementation levels.

Another limitation is that we did not measure the number of total downloads nor track downloads or app usage to individual students. This decision was made to preserve the anonymity of students and trust between the administration and student body. Despite this limitation, the number of mistreatment reports has remained nearly twice as high as mistreatment reports pre-implementation, when reporting was done through direct outreach to Student Affairs Deans. We are therefore confident that the app is being downloaded and utilized regularly by our students and is the primary contributor to improving awareness and utilization of our mistreatment policies and procedures.

The final limitation of the app is the inability in its current form to host two-way communication with the reporter without compromising anonymity. This is especially salient when follow-up questions are required to investigate or act upon a student’s report. We are working to add this feature in a future update of the app. We do, however, ask students to provide demographic data (e.g., class year, incident site, rotation/course), which we use to inform broader patterns should other reports of mistreatment emerge.

Future work will also focus on increasing consistency in follow-up processes and outcomes after a student reports mistreatment. To accomplish this, we have formed a group of select leaders, including representatives from Student Affairs, Office of Diversity, Equity, and Inclusion, Clinical Education, and Assessment to standardize our approach to addressing mistreatment reports. This group will work together to aggregate the mistreatment report data to be shared with students, faculty, and administration annually. We anticipate that data collected from the app will have a lasting impact on the quality and consistency of our medical students’ experiences.

Overall, we have found that reaching students in familiar and easily accessible mode(s) of communication is effective and can catalyze lasting change.^11^ This strategy can be applied to many aspects of medical education and is replicable at other institutions.

## Data Availability

The data that support the findings of this study are available from the corresponding author, SB, upon reasonable request.
